# A co-produced mixed methods protocol: Exploring perceptions of oral health care and quality of life in people with mental health conditions

**DOI:** 10.1371/journal.pone.0313983

**Published:** 2025-01-16

**Authors:** Michelle R. Zechner, Yuane Jia, Naja Hill, Ann Kasper, Jill York, Vaishali Singhal, Pamela Rothpletz-Puglia

**Affiliations:** 1 Department of Psychiatric Rehabilitation and Counseling Professions, Rutgers School of Health Professions, Piscataway, New Jersey, United States of America; 2 Department of Interdisciplinary Studies, Rutgers School of Health Professions, Newark, New Jersey, United States of America; 3 Kasper Connects, New York City, New York, United States of America; 4 Department of Community Health, Rutgers Health School of Dental Medicine, Newark, New Jersey, United States of America; Shahid Beheshti University of Medical Sciences School of Dentistry, ISLAMIC REPUBLIC OF IRAN

## Abstract

This planned mixed methods protocol is designed to explore oral health care for individuals living with serious mental health conditions (SMHC). It was co-produced by academics, people with lived experience of mental health conditions, and oral and mental health clinicians. The study seeks to explore oral health quality of life predictors and oral health care experiences of people diagnosed with serious mental health conditions (e.g. schizophrenia, bipolar disorder, major depression and general anxiety disorder) about their oral health care experiences. The research research will generate recommendations for creating positive oral healthcare experiences for people living with SMHC. Research co-production with people diagnosed with mental health conditions is a recommended strategy to improve the utility and relevance of health research, as well as empower a disenfranchised population.

## 1. Introduction

### 1.1 Rationale

People diagnosed with serious mental health conditions (SMHC) experience significant health disparities. While the rates of dental challenges and barriers to oral care for people living with SMHC have been previously explored [1], less is understood about how to engage people living with SMHC into dental care, how people studied perceive their treatment in oral care and the factors that influence oral health quality of life. Barriers to oral health for people living with SMHC include experiences of being misunderstood or treated unequally by oral health providers and dental clinic staff, experiences of stigma, challenging life experiences, previous negative experiences, and a lack of adapted and accessible information for their situations [1–4]. More research is needed to understand oral health quality of life in people living with SMHC and understand experiences and recommendations from the population to overcome significant challenges [2].

There is a gap in the literature identifying research-driven guidelines and education to engage people with SMHC in oral care and a lack of understanding about the oral care experiences of people living with SMHC, as well as oral health providers feeling unprepared to work with the population [5]. Gathering experiences and recommendations can assist with the development of guidelines for positive oral health experiences and education for oral health providers and people with SMHC.

This study seeks to gain information about oral health experiences and oral quality of life from people with SMHC to inform the development of positive oral health experience guidelines and professional development and community educational products.

### 1.2 Need for input from service recipients

People diagnosed with SMHC face intersecting barriers to comprehensive oral health care related to the social determinants of health. Some examples of these determinants include inadequate dental insurance limiting types of procedures (e.g. emphasis on extractions over dental restoration or implants), lack of funding for dental care of publicly insured individuals, few in-network dental providers, inadequate transportation, and previous negative dental or medical experiences. In addition, the personal experiences of reduced oral health literacy, likelihood of previous trauma exposure, lifetime experience of poverty or low income, historically under-served by medical providers and experiences of stigma present barriers to accessing dental care [1, 3, 4, 6–8]. People with mental health conditions also note discouraging experiences with oral health providers and clinics unprepared to provide oral health care to people who have experienced previous traumatic experiences [4, 5].

Given these complex overlapping issues, it is unsurprising that people with mental health conditions may not seek out or receive oral health treatment. In order to overcome challenges to accessing and receiving care, researchers must solicit input from people with SMHC about strategies to better engage and overcome existing barriers to comprehensive oral health care. Gathering diverse lived experiences (e.g. dental and mental health) from people with mental health conditions can address practical issues, perceptions of care, and identify advocacy work that may be needed. Seeking and receiving oral health care is influenced by the impact of mental health and previous health service experiences. Building meaningful partnerships with people diagnosed with SMHC as research collaborators is crucial to understanding experiences and identifying recommendations for effective and positive oral health care for the population.

### 1.3 Co-production of research with people with SMHC

Co-production of research with people with SMHC suggests a meaningful collaboration between academics and people with lived experience of a serious mental health condition to design and conduct research projects [9]. In these models, the populations being studied are involved in the development, refinement, improvements and process of the research [10]. Co-produced research can improve quality of projects, empower persons with lived experience, ensure meaning of projects and offer greater relevance to all stakeholders [9–11].

The current study protocol began with academic researchers applying for and receiving grant funding to study the oral health experiences of people with mental health conditions with oral care to develop guidelines and education for positive oral health care. The study procedures incorporated two primary strategies to gather valuable feedback from people with SMHC to help shape the research protocol and interview guide. A Lived Experience Consultant was hired to participate in team meetings, maintain a lived experience dialogue about effective procedures and outcomes, and offer feedback and input into the study procedures and process. Additionally, the researchers convened a 12-member Lived Experience Advisory Board with experts of lived experience from a variety of geographic regions in the United States. Techniques to engage people with lived experience in the process included developing a climate of mutual respect and appreciation for lived experience knowledge, utilizing active listening, seeking input from all voices during meetings, encouraging communication through multiple modalities, emphasizing project goals of education and improvement of oral health in people with SMHC and the sharing of the research team’s personal aspects during interactions (e.g. explaining their own experiences of oral health care challenges). The Lived Experience Consultant assisted in facilitating the online advisory board meeting using the techniques and peer culture-influenced meeting skills that kept the discussion fluid and ensured the inclusivity of all voices. Most members of the board were fully engaged and participated through sharing their reflections orally or by chat. The meeting chat was saved for later review and guidance. These strategies and processes are similar to those reported previously in co-production literature (e.g. navigating power, multi-directional learning, offering space and connecting through vulnerability) [9].

As a result of the application of co-production techniques the project expanded in scope to include data collection of oral health quality of life and corresponding social determinants of health. The convened lived experienced experts also voiced significant concern that a qualitative study of 30 persons may not represent the wide range of oral health experiences the population may face. Therefore, our initial procedure plan, which included only qualitative interviews with 30 people, was modified and expanded to address these concerns. The protocol was revised to utilize a mixed methods technique to collect social determinants and oral health quality of life information from a larger sample (n = 150) during *Phase 1*. The responses will then be sorted into two groups: people with high and low oral health quality of life as determined by a validated tool, and purposive sampling will be used to recruit people with diverse oral health experiences for interviews (n = 30) in *Phase 2* of the study. This re-working of the data collection and recruitment for diverse experiences made the project more relevant to people with SMHC and empowered the voice of people living with mental health conditions. This will ensure a more robust collection of recommendations for the future work of developing positive oral health experience guidelines.

### 1.4. Research questions

The co-production process led to the refinement of the research questions that the protocol is designed to answer:

1. What are the relationships between oral health quality of life, social determinants and other demographic factors for people diagnosed with SMHC such as schizophrenia, schizo-affective disorder, bipolar disorder, major depression, and general anxiety disorder?
2. What is the oral care experience for people living with SMHC?
3. How do people with SMHC recommend that dental care providers create a positive dental care experience?

### 1.5. New evidence offered

Studies exploring oral health quality of life and dental experiences of people living with mental health conditions are limited [4, 12]. Research combining oral, social and behavioral health determinants of health is vital to understand best how to engage the population in oral health care and ultimately improve oral health outcomes [12]. The current study offers strategies to meaningfully engage people with SMHC in oral health research. This will provide new information about oral health and understanding of the relationships between oral health, quality of life, social determinants of health and dental experiences. As a result of the work there will be a greater understanding of the relationships between the social determinants of health and experiences that influence oral health quality of life for people living with SMHC. Lastly, the proposed project will garner recommendations from diverse people living with SMHC to inform draft stakeholder recommendations on creating a positive oral health experience for the population.

## 2. Materials and methods

### 2.1. Aims and objectives

This mixed methods explanatory sequential study protocol aims to understand oral health quality of life and oral health care experiences of people living with SMHC (e.g. schizophrenia, schizoaffective disorder, bipolar disorder, major depression and general anxiety disorder) to generate recommendations for creating a positive oral health care experience for the population in question. A secondary aim is to identify predictors of oral health quality of life in people with mental health conditions. Participants will be recruited through community mental health services for people with SMHC, peer training electronic mailing list distribution lists, advocacy organizations and snowball sampling to complete an online survey (n = 150) by self-report or may choose to complete it via proctor who will read the questions aloud and complete the online survey. Inclusion criteria for the study will/must include a person self-reporting being diagnosed with a serious mental health condition (e.g. schizophrenia, schizo-affective disorder, bipolar disorder, depression, and general anxiety disorder), fluency in English, living in the community, and willing to discuss experiences and recommendations for oral health providers. Exclusion criteria includes: persons with a legal guardian.

An explanatory sequential mixed methods design will be used to learn more about oral health-related quality of life for people living with SMHC using a two-phase approach. This study was approved in a two-step process by the New Jersey Alliance for Clinical and Translational Science, Scientific Review Board and by the authors’ university Scientific Review Board the Rutgers (Project ID #Pro2024000700) to begin data collection from 6/28/24 through 5/31/25. Written informed consent will be obtained from all participants of the study.

#### 2.1.1. Phase 1 (surveys)

The *Phase 1* data collection includes demographic data and health related quality of life measures. A researcher constructed demographic survey was developed based upon previously validated tools including the National Health and Nutrition Examination Survey (available at https://www.cdc.gov/nchs/nhanes/index.htm) [13], The Centers for Disease Control and Prevention’s Healthy Days Measures (available at www.archive.cdc.gov) [14], Kaiser Permanente’s Your Current Life Situation (available at www.kaiserpermanente.org) (YCLS) [15] and The Xerostomia Inventory [16]. The Oral Health Impact Profile- Brief Survey (OHIP-14) [17] is a 14 question self-report survey to identify oral health issues present in the past year. It has been validated and used in people with SMHC [12, 16, 17] to assess oral health quality of life. Reliability alpha values for the tool range from .88 in a study of non-clinical populations [15] to .93 in a study of people with mental health conditions [12]. Lastly, the Centers for Disease Control HRQOL-4 “Healthy Days Measure” [18], is a 4-item validated tool measuring health related quality of life which has been widely used in population health studies, including people with SMHC [18–21]. Each question results in a number of self-report days of ill health in the past 30 days related to physical or mental health issues [19]. Mean values of unhealthy days are calculated to represent the quality of health, and a sum mean score of unhealthy days are often presented to represent health quality of life [18].

#### 2.1.2. Phase 2 (interviews)

Recruitment for *Phase 2 (Interviews)* will be purposely sampled from *Phase 1* participants who indicate interest in sharing their experiences in *Phase 2* of the study based upon their oral health related quality of life survey results. Efforts will be made to ensure representativeness of mental health conditions and demographic attributes and recency of last dental visit. The qualitative interviews will take place in an online meeting platform to allow recruitment from a wide geographical area to capture diverse individual experiences. The Principle Investigator (PI) and research team will purposively sample and invite 30 participants from the Phase 1 pool of survey participants to be interviewed based on oral health quality of life scores, recency of dental visit and representativeness of various mental health conditions and demographic attributes. Data Analysis.

*Phase 1* quantitative data analysis will include data screening and assumptions check, descriptive statistics (e.g., mean, standard deviation for continuous variables, frequency and percentage for categorical variables) for all the variables in the sample. Bivariate correlations among all the scale measures will be calculated using Pearson/Spearman correlation. T test or ANOVA will be used to explore the relationship between OHQoL/health related QoL and the categorical demographic variables. Regression analysis will be conducted to predict higher and lower scores on OHRQoL using demographic variables, physical health, mental health diagnosis, and health related QoL.

*Phase 2* data analysis will utilize an interpretive phenomenological approach for in-depth learning from 25–30 participants diagnosed with SMHC about the experience of oral care. Interpretive phenomenology is a methodological tradition in qualitative research when the aim is to learn about the emotions and personal meanings surrounding an experience." [22]. Rich descriptions of memorable dental care experiences (positive or negative) will be captured in interviews using the critical incident technique [23, 24]. The critical incident technique is a strategy employed to enable participants to tell a complete story, including the context or situation of the oral care experience, the interactions, procedures, or events that took place during the experience, and the outcome of the experience. We will also specifically ask participants for ways to improve dental care experiences.

The estimated number of interviews needed to reach theme saturation is 25; however, we will invite 30 to account for possible attrition. We anticipate that code and meaning saturation (information power) will occur with this sample size [19, 25]. The interviewers do not identify as having mental health lived experience themselves, however, have had training and experience conducting interviews with people diagnosed with mental health conditions.

The data collected in *Phase 2* will be generated from an interview guide with open-ended questions. The questions elicit information about oral health experiences, memorable oral health experiences, and recommendations for improving oral health care experiences (See S1 File Interview Guide).

Interpretive phenomenological analysis strategies will be used to formulate experiential statements, locate connections and patterns, compile experiential themes, and conduct cross-participant analysis. Our research team, experienced with qualitative research, will employ several strategies to ensure the research’s trustworthiness, such as maintaining a transparent data collection and analysis process, reaching researcher consensus on thematic statements, and member-checking with the experts on the Lived Experience Advisory Board about the interpreted thematic statements [26]. This process will culminate in a preliminary set of guidelines for dental care providers and other stakeholders from people living with SMHC.

#### 2.1.3. Data management plan

*Phase 1* completed online survey data will be stored on university HIPAA compliant survey software. Participants may choose to complete the survey anonymously without offering their names or other identifying information. Participants may opt into Phase 2 (interviews) by choosing to share their first name and last initial, email address and telephone number after answering the initial online survey. Identifiable data will be replaced with a study number stored separately from the other survey data. This linking information will be kept until the end of Phase 2 data collection (e.g. interview and review of interview summary), and all identifying information will be removed and destroyed. The key code will be stored on password protected computers and files.

Participants can request compensation through a separate survey, at which point they are requested to share their names and contact information to receive a $10 gift card. They may opt out of this procedure. This information will be stored in a separate online survey from research data. Upon completion of data collection, identifying information will be replaced with research-assigned numeric identifiers and all names will be removed from the dataset. Once enrollment is completed, all personal identification collected for the gift card information will be deleted and destroyed.

*Phase 2* interview data will be recorded using university encrypted web conferencing audio recording. The audio recording will be transcribed via NVivo’s AI software, and once completed, audio recordings will be deleted. Transcripts will be deidentified. Participants will be compensated with a $50 electronic gift card. They may opt out of this procedure. This information will be stored separately from the transcripts, and there will be no linking information.

## 3. Results

The proposed study is currently recruiting for participants in Phase 1. Phase 2 recruitment will follow the completion of the Phase 1 data collection. Summary findings will be made publicly available on a project-specific website, and de-identified data will be stored in the public data repository of Interuniversity Consortium for Political and Social Research (ICPSR).

## 4. Discussion

The current study protocol will seek to understand the predictors of oral health quality of life, explore relationships of oral health quality of life with social determinants and other factors, and identify the experiences and recommendations for creating positive oral health experiences in care. The planned study included the involvement of stakeholders in oral health care, including people with SMHC, oral health providers (e.g. dentists, dental assistants, dental hygienists) and mental health providers (e.g. social workers, psychiatric rehabilitation practitioners and administrators) through the use of advisory boards and an inter-professional investigator team (e.g. psychiatric rehabilitation, dentist, dietician and statistician).

There are some limitations to the current planned study. The study is limited to English-speaking and reliant on some level of computer literacy, which limits the generalizability of findings. Additionally, the cross-sectional design only seeks data collection from one time-point, limiting the ability to establish causality or track changes over time. Further, the study’s sample might not fully represent the diverse population of persons with lived experience of SMHC and a larger sample is needed if sub-group analysis is planned. Efforts are being made to solicit participants across geographic location and setting type (e.g. peer provided services, housing programs, outpatient mental health and justice involvement) to address this limitation. Participants’ responses about their oral health experiences may be influenced by recall bias or social desirability. Findings may not apply universally due to variations in cultural norms, healthcare systems, and access to oral health services. Additionally, engaging people diagnosed with mental health conditions in research can be difficult due to stigma or lack of trust in researchers. Future studies should consider utilizing lived experience experts to interview their peers to ensure linguistically accurate answers from the persons being studied, and that cross-cultural contexts (dental medical culture and peer cultures) in dental offices are understood at that level, and to overcome trust issues. To increase recruitment and engage our community, snowball sampling will be applied which might not cover represent true diversity of experience.

This study is the first in a series of projects in a larger inter-professional federal grant that aims to create educational materials for oral health providers and people living with SMHC to promote positive oral health experiences for people living with SMHC. Findings will inform the development of preliminary recommendations for a Delphi Study with stakeholders to formulate Guidelines for Positive Oral Health Experiences, and educational materials for oral health providers and people with SMHC. In addition, these materials will be used to create an online university-sponsored micro-credential badge available through the university to oral health providers.

## 5. Conclusion

Co-production of protocols and research about oral health care in people living with SMHC can ensure meaningful and collaborative outcomes. Oral health and oral health quality of life are dramatically worse in people living with mental health conditions compared to the general public due to the complexity of intersecting social determinants of health, experiences with mental and oral health systems, and mental health symptoms [27, 28]. Researchers must address these areas sensitively and in partnership with people living with mental health conditions. Our protocol development significantly involved the Lived Experience Board members and our Lived Experience Consultant to shape and influence the development of our interview guide and to strengthen our focus on oral health quality of life. This ultimately led to a more robust data collection and a two-phase approach. Our lived experience collaborators will also help understand and contextualize our findings. Further, the input from a Practitioner Advisory Board consisting of oral and mental health providers to inform the project (e.g. dentists, dental hygienists, dental assistants, clinical supervisors and executive directors from programs serving people with mental health conditions), ensures a practical model.

More work is needed to study best practice co-production meeting techniques in health sciences research, which utilize the lived experience culture and trauma-informed meeting practices [9]. The lived experience peer culture and traditional academic cultures are considerably different. In our example, the Lived Experience Consultant actively worked to bridge these cultural differences during meetings. Some experts by experience do not have academic experience and feel intimidated to join fully in co-production. The balance of power can intimidate peers. The Lived Experienced Consultant understood some of the fears and was able to encourage lived experience participants to speak up and offered encouraging feedback for the comments they added. The academic team must be proactive in identifying lived experience experts who can help to bridge these divides, as people may feel hesitant to offer authentic feedback, that is extremely valuable to ensure the success of any project studying people with SMHC.

Previous examples of co-production utilized broad collaborative approaches, which began with a collaboration between researchers and people with lived experience of serious mental illness developing research questions, submitting grants together and developing educational materials and processes [9, 29]. Co-production examples include educational materials to improve oral health literacy and oral health recovery for people living with SMHC [30, 31].

The topic of oral health and its impact on quality of life brought up strong emotions with the people with lived experience in the lived experience advisory group. Oral health impacts a person’s quality of life in significant ways, such as the ability to chew or speak; untreated dental issues cause physical pain, shame, or embarrassment. These issues may ultimately cause challenges with emotional, social, or vocational wellness [31]. Additionally, many of our lived experience collaborators shared their experiences of dental providers choosing to pull teeth rather than offering choices for less invasive dental treatment. This brought about a sense of marginalization and discrimination. Through co-production and collaboration with people with SMHC, meaningful research and interventions can be developed, which can improve the oral health quality of life of this under-served population.

## Supporting information

S1 File Exploring dental health for people living with mental health conditions *qualitative interview guide*. (PDF)
