# Supplementary material for: A co-produced mixed methods protocol: Exploring perceptions of oral health care and quality of life in people with mental health conditions
Source: PLoS One. 2025 Jan 16;20(1):e0313983. doi: 10.1371/journal.pone.0313983 (PMC11737713; doi:10.1371/journal.pone.0313983)
Supplement: S1 File — (PDF) [file pone.0313983.s001.pdf]

Exploring Dental Health for People Living with Mental Health Conditions
Qualitative Interview Guide

1. Where and when do you go for dental care services?
    a. How do access issues impact your ability to visit the dentist when you need or want to?
    b. How often do you go for dental care each year?
    c. In what ways has your routine for dental visits changed or stayed the

2. In general, how does the experience of going to the dentist make you feel?
    a. How long does it take to get an appointment?
    b. How is it for you at a dentist visit?
    c. How comfortable were you in the dental office space/physical environment?

3. Please tell me about a memorable visit to the dentist.
    a. Why was the appointment scheduled?
    d. Whait are the reason for the visit (cleaning, fillings, extractions, gum care, implants, dentures, other procedures, etc.)
    b. What happened during the visit?
        i. What did the dental care professional do? What did you do?
    c. What was the outcome of the visit?
        i. How did you feel about the visit?

4. Depending on the scenario the participant provides in question 3, ask how the experience compares to other visits, how it impacted their dental care, and how the visit could have been better or different.
    a. How did this experience impact your future dental care?
    b. How does the experience you shared compare to what you consider typical?
        i. If not, how is it different from other visits to the dentist?
    c. What could the dental care professional do differently?
    d. What, if anything, do you think you might have done differently in the situation?

5. What would help you with your dental care?
    a. What is important to you when dealing with a dentist or other oral health care provider?
    b. What would help you get to your dentist appointments and take good care of your teeth? Is there anything preventing you from good dental care?
    c. What does optimal dental care look like to you?

6. What advice do you have for dental care providers about caring for people diagnosed with mental health conditions?
    a. How can dental care providers create a positive experience for people diagnosed with a mental health condition?
